# Questioning the answer: questioning style, choice and self-determination in interactions with young people with intellectual disabilities*

**DOI:** 10.1111/j.1467-9566.2009.01223.x

**Published:** 2010-03

**Authors:** Alison Pilnick, Jennifer Clegg, Elizabeth Murphy, Kathryn Almack

**Affiliations:** 1 School of Sociology and Social Policy, University of Nottingham; 2 University of Nottingham and Nottinghamshire Healthcare NHS Trust; 3 College of Social Science, University of Leicester; 4 School of Nursing, University of Nottingham

**Keywords:** intellectual disability, choice, self-determination, conversation analysis

## Abstract

For young people with intellectual disabilities (ID), the transition from children's to adult services has long been recognised as a challenging move. One of the aims of the White Paper *Valuing People* (2001) was to address some of the problems associated with this transition. This paper reports on data from a project which examines the impact of these service changes, and the ways in which transition is negotiated by carers, professionals and users. It presents a conversation analysis of eight tape-recorded formal review meetings at which transition to adult services is discussed. It takes as its starting point the existing interactional work on ID and the way in which this demonstrates the effects of the local and contextual specifics of particular kinds of interaction on the eventual outcomes (*e.g*. Rapley 2004, Antaki 2001, Maynard and Marlaire 1992). We show that an attempt to allow self-determination in the context of transitions can paradoxically result in undermining user choice and control. We also argue that, while a rule-based approach to practice may offer moral clarity for professionals, it can result in interactional and practical difficulties which cannot be easily reconciled.

## Introduction

For young people with intellectual disabilities (ID) and their carers, transition from children's to adult services has long been recognised as a challenging issue. The young person has to leave behind the package of care with which they have become familiar, and a new package has to be negotiated. The 2001 White Paper, *Valuing People*, specifically addresses problems with transition to adult services in its recommended service changes. The data presented in this paper are drawn from a larger project which aimed to examine the impact of these changes, and to study how users, carers, professionals and service providers negotiate access to services for young people with ID.

To examine the impact of ‘Valuing People’ on transitions, it needs to be set in its wider policy and political context. Over recent years a strong normalising discourse has emerged in relation to people with intellectual disabilities, and as May and Simpson (2003) note, this presents self-determination as an inalienable right. Murphy *et al.* (forthcoming) identify how this shift in thought is embodied in *Valuing People*, which lays out four key principles which should underpin services for adult intellectual disability: rights, independence, choice and inclusion. They also note that the White Paper contains no suggestion that impaired capacity should constrain self-determination.

This focus on normality and empowerment is, as Fisher (2008) points out, consistent with a neo-liberal concept of healthy citizenship which has been a key aspect of New Labour policy. As she puts it, ‘The intention is that excluded groups, such as…people with disabilities…should be brought into the realm of ‘mainstream’ society constructed around notions of independence and paid work’ (2007: 284). However, she argues that a focus on empowerment tends to exaggerate voluntarism and to underplay constraints (Fisher 2008), and cites Rose (1999) who suggests that within this discourse there is little space for any interpretation of empowerment that is not equated with individual self-sufficiency.

The continuing influence of this neo-liberal underpinning can be seen across policy reform throughout health and social care services. The 2008 Green Paper *No-one Written Off: Reforming Welfare to Reward Responsibility* lays out plans for work incentives to ‘create a system that rewards responsibility’ and ‘enables people to become the authors of their own lives’ (2008: 11). The subsequent 2008 White Paper *Raising Expectations and Increasing Support: Reforming Welfare for the Future* sets out a vision for a welfare state where ‘everyone is given the help they need to get back to work, matched by an expectation that they take that support’ (2008: 9). In wider policy terms, then, it seems that empowerment increasingly translates explicitly and directly into paid employment. In relation to this, the 2008 White Paper specifically addresses those with disabilities, stating that ‘Our vision is a society where there is equality for disabled people’ (2008: 83), and where only the ‘most disabled’ would form part of a group where there was no requirement for any work-related activity. Clearly, the equality that is envisaged translates not just into addressing issues of discrimination, but also into an equal responsibility for those with disabilities to be economically active.

Evidently, any notion of ‘normality’ which is contingent on independence through self-sufficiency, or on empowerment through paid employment, is far from straightforward when applied to young adults with learning disabilities. To some extent this has been recognised elsewhere in policy: the *Mental Capacity Act (England and Wales)* (2005), which came into effect during 2007, acknowledges that more than two million people, including some with intellectual disabilities, lack capacity to make at least some decisions about their lives. In these cases substitute decisions may be made, but the guiding principle is that these substitute decisions should uphold the person's best interests. This of course leads to the question of how these best interests are to be determined. The White Paper *Reforming Welfare* (2008), whilst making clear the responsibilities of welfare recipients, considers this issue specifically. Its solution is an extended version of the personalised service that is emphasised throughout the reforms, stating that ‘We want to support claimants to choose their own programme of work-related activity, as we recognise they know their own individual circumstances, needs and goals best. However, a minority may need more guidance.’ (2008: 90–91). Despite this recognition, there is no detail provided as to the form this guidance might take or how directive it might ultimately be.

As this brief review has shown, there is no clear consensus as to the application of principles of self-determination and choice to adults with intellectual disabilities. While *Valuing People* lays out a vision of universal self-determination through its four key guidelines, it has been recognised elsewhere that the application of this may be problematic (Burton and Kagan 2006, Cumella 2008), and this problem is highlighted in more recent legislation and policy (*Mental Capacity Act* 2005, *Reforming Welfare* 2008). Accordingly, this paper attempts to shed some light on how these principles are *practically* applied, by examining in detail interactions between young adults with ID and the professionals involved in their care, in a scenario where decisions affecting the young person's future must be made.

## Interactional research into ID

Over recent years there has been a significant amount of research conducted into ID from an interactional perspective. Authors such as Rapley and Antaki (Rapley 2004, Antaki 1999, 2001, Antaki and Rapley 1987) have examined interactions involving people with ID in careful detail. This work has often been presented as a critique to the mainstream psychological literature on ID, which Rapley (2004) argues represents an attempt to account for the conduct of people with ID in terms of individual or dispositional characteristics rather than contingent ones – in other words that the conduct attaches to the person, rather than the circumstance. However, as work by Marlaire and Maynard (1990) and Maynard and Marlaire (1992) shows, many of the tools and tests used to assess persons with ID have an interactional basis, and the results of these may be as much dependent on the way in which questions are asked as on the person answering them. This critique has been particularly strong in relation to the phenomenon of ‘acquiescence bias’. Put simply, acquiescence bias can be described from the perspective of the person with ID as ‘if in doubt, say yes’ (Sigelman *et al.* 1981); in other words, it represents a tendency to respond in the affirmative or to agree with the interviewer, regardless of the content of a question. Since the 1980s the notion of a dispositional acquiescence bias among people with ID has become widely held to be true.

Through detailed analysis of data collected in test situations, Rapley shows that what might at first glance look like acquiescence bias may in fact be a perfectly competent interactional response to the contingencies of a particular situation – what he calls ‘pseudo acquiescence’. The example below comes from his data (2004: 93).


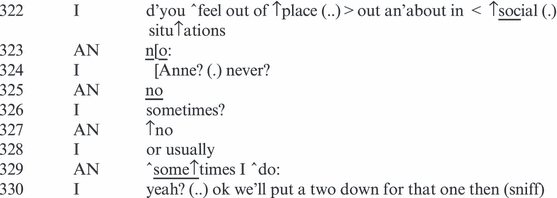


In this instance then, Anne's initial response of ‘no’ is pursued by the interviewer, who needs to record a ‘standard’ answer (never, sometimes, usually) for the survey. After the initial pursuit (‘never?’) Anne holds firm, and continues to do so while ‘sometimes?’ is offered as a possibility. It is only when a third pursuit occurs (‘or usually’) that Anne modifies her initial answer and reformulates it in terms of a standard response. However, as Rapley notes, it is highly debateable whether this represents submissive acquiescence on Anne's part, or whether it is contingent both on the interviewer who doesn't accept what is an interactionally adequate answer as acceptable for these purposes, and Anne's subsequent and related recognition that this is a particular kind of question needing a particular kind of response.

Much of the interactional work on intellectual disability has been carried out in very specific interactional settings, involving test questions, formal surveys etc, where what is considered to be an appropriate response must often take a very specific form (as in the case of ‘never’ rather than ‘no’ in the example above). Rapley (2004) goes on to explore the transferability of some of his findings in less formal settings, but until recently, there has been little detailed work on the interaction of people with ID in mundane settings. Recent work by Finlay and colleagues (Finlay, Antaki and Walton 2008, Finlay, Walton and Antaki 2008, Finlay *et al.* 2008) has addressed this, by examining video data of day-to-day interactions between people with ID and care staff, looking at for example how games are played (Finlay *et al*. 2008) or how refusals (such as in the context of routine weighing) are achieved (Finlay, Antaki and Walton 2008). One theme that this has in common with Rapley's work is that it focuses on the competencies that people with ID can and do display in different types of interaction, and the ways in which these may be capitalised upon to promote choice and self-determination. However, this most recent work also addresses the gap between policy goals and practice (Finlay, Walton and Antaki 2008), noting that ‘Empowerment is not just about choosing to take this type of support rather than that…but it is about what happens between people moment by moment, in the mundane details of everyday interaction’ (2008: 350). The authors go on to suggest that the level of choice or control that a person with ID in a residential setting has can only be assessed by detailed observation, noting such apparently mundane things as whether they can choose when and how much to eat. We share an orientation to examining the details of interaction in order to shed light on the practical difficulties of policy implementation for people with ID, but apply it here to a rather different setting.

## Methods

The project was funded by the Big Lottery Fund in association with Nottingham Mencap. The study cohort focuses on young people (aged 18/19) leaving special schools in 2004/5 within two related English localities: a town, and its related suburbs and villages. Transition staff identified eligible families for participation. Of the 44 young people who left school over this period and were supported by specialist staff, 28 participated in the study. Four declined to take part. Of the remaining 12, confidentiality means it is not known how many were excluded by transitions staff as ineligible and how many did not respond. Families who did respond were visited by the researchers who explained the project in detail and negotiated consent. Approval from an NHS Research Ethics Committee was obtained. Carers and transition workers gave written consent, and the young people gave verbal consent. The project had a longitudinal design involving repeat interviews with carers, Transition Co-ordinators, Disabled Persons Act workers, Connexions Personal Advisers, teachers and other service providers, for example representatives of specialist services for autism. Transition Co-ordinators are employed by local authority social services departments, and Connexions Personal Advisers are employed by Connexions, a centrally funded agency which evolved from the Careers Guidance service.

The research also included individual interviews or discussion groups with the young people (where possible), and observation and recording of interactions during meetings. This paper focuses on audio-tape recorded data collected from eight multi-party meetings: four Transition Planning Meetings, and four Leaver Review Meetings. It is a requirement that Transition Planning Meetings are organised annually by the school which the young person attends from Year 9 onwards. These meetings document what the person wants to achieve and what support they need to live as independently as possible. The meetings we observed and recorded were the last of these annual meetings before leaving school. Such meetings are intended to cover all aspects of life: education, employment, housing, health, transport and leisure. Where possible, the young person, their parent/carers and representatives of all agencies involved with them are present.

Leaver Review Meetings occur differently in different localities. They are usually organised by the Connexions service. They review progress (both educationally and with plans for future activities), are held a number of times during the young person's final year at school, and generally involve fewer people from outside agencies. Both Transition Planning Meetings and Leaver Review Meetings took place in the educational setting that the young person was currently attending. In all cases a teacher from the school chaired the meetings and took responsibility for the agenda, with the exception of one case where a Social Services team manager was asked by the teacher to act as chair. More detail on the young people who were the focus of these eight meetings and their circumstances are included in Table 1 below. The data presented here are analysed using conversation analysis (CA). This is an approach that examines the moment-to-moment organisation of interaction through talk, and the way in which each utterance is both context shaped (organised in the light of the prior action) and context renewing (framing the next action) (Heritage 1984). It is distinctive in providing the opportunity to focus on members’ own displayed orientations to social action. (For a comprehensive introduction to the approach, see ten Have (2007)).

**Table 1:** Sample details

Number & pseudonym	Type of meeting	Persons present	Level of disability	Communication (as described by parents)
01 Stephen	Leaver's review	Teacher, Social Services Team Manager, Day Services Co-ordinator, Father, Mother, Two Day Service Workers.	moderate	Communicates in short sentences, understands others.
09 Andrew	Leaver's review	Teacher, Transitions Co-ordinator, Agency Worker, Agency Manager, Team Leader at Day Service, Key Worker at Day Service, Mother, Consultant psychologist, Speech Therapist.	profound and multiple	No verbal communication. Limited understanding.
10 Louise	Leaver's review	Teacher, Transitions Co-ordinator, Day Services Co-ordinator, Father, Mother.	moderate	Communicates in short sentences, understands others.
20 Helen	Leaver's review	Teacher, Transitions Co-ordinator, Mother, Father, Key worker at Day Service, Day Service Worker.	severe	Limited verbal communication. Uses Makaton. Some understanding of others.
26 Sam (male)	Transition	Head Teacher, 6th Form teacher, Father, Specialist worker linked to Day Service, Day Service Worker, Manager of Adult short break service, Transitions Co-ordinator.	moderate	Communicates in short sentences, understands others.
29 Adam	Transition	Head of Sixth Form, Key Teaching Assistant, Mother, Connexions Worker, Transitions Nurse, Transitions Co-ordinator.	profound and multiple	Almost no verbal communication. Limited understanding.
30 Alec	Transition	Head of Sixth Form, Mother, Transitions Nurse, Connexions Worker, Transitions Co-ordinator.	moderate/severe	Communicates in short phrases or single words. Understands others.
31 Sally	Transition	Head of Sixth Form, Mother, Transitions Nurse, Transitions Co-ordinator, Connexions Worker, Worker at Children's Short Break Service.	moderate/severe	Communicates in short sentences. Some understanding.

As the brief review above has shown, CA has already been applied to a number of scenarios in the ID field. The data presented here, however, differ from the data contained in previously published interactional research in two important respects. The first is in the level of disability of the participants in the study. With the exception of very recent work by Finlay and colleagues (Finlay, Antaki and Walton 2008, Finlay Walton and Antaki 2008, Finlay *et al*. 2008), many of the studies described above examine interaction involving people with mild to moderate ID. In order to participate in verbal tests, survey interviews *etc*, they must have a relatively high level of verbal communication skills. The young people in the study sample here had been judged to have moderate to profound ID. The second issue is that the context here is neither mundane (as in many care home interactions) nor fixed (as in survey administration) but falls somewhere in between. Transition Meetings and Review Meetings are more open in format than the administration of a standard list of questions, but they nonetheless have agendas that must be covered and endpoints that must be reached, which differentiates them from many more casual interactions with care staff. Whilst there is some work on service-user meetings in residential and day services (Antaki *et al*. 2006, Jingree *et al*. 2006), these are less formalised than Transition and Review Meetings, and bring together a number of service users rather than one individual who will be the focus of the meeting.

## Analysis

The analysis that follows is divided into four sections. We begin by examining the ways in which professionals explicitly attempt to place the young people as active participants at the centre of these meetings. We then move to consider aspects of questioning style as a possible interactional impediment to young people's participation. Thirdly, we examine the consequences of what is deemed to be inadequate or inappropriate participation. Lastly, we explore a case where the limits of self-determination in this setting become apparent.

## Delivering choice and control

In any consideration of people with ID, the issues of choice and control are never far from the forefront. A detailed discussion of these concepts as they apply to ID is beyond the scope of this paper, but as Wilson *et al.* (2008) note, these principles are so prominent partly through a strong desire for ID professionals to distinguish themselves from past practice, in particular institutionalisation and infantilisation. Redley and Weinberg (2007) describe how ‘recent policy initiatives have moved decisively towards empowering learning disabled citizens, recognising ability over disability, and promoting people's political empowerment and voice in the design of public services’ (2007: 767). Finlay, Walton and Antaki (2008) note how policy guidance tends to present the expression of preference as unproblematic, and to focus instead on the difficulties of translating preference into action. However, as we mentioned at the outset, and as many authors have recognised (*e.g*. Burton and Kagan 2006, Swenson 2008), the difficulty becomes evident in how to apply these concepts in a meaningful way in this context, so that exercising control and making choices can be apparent in daily life for people with ID. There is certainly no easy answer to this difficulty, as the interactional evidence shows.

As might be expected, given the clear policy agenda, in the data taken from these meetings, there are many formal and explicit references to the right of the young person under discussion to be placed at the centre of the process and to be given an opportunity to direct the agenda. The meeting below begins with a statement of purpose that is formally addressed to the young person, though it is available as a resource to all those present. This statement also clearly allocates primary speaker and listener roles ‘And we're all going to hear (.) and you're going to talk to us...’ (lines 31-32). (In the data that follow, young people are referred to by pseudonyms, and parents or carers by their relationship to the young person (*e.g*. ‘mother’.) SPTeach denotes a special education teacher, SSTM a Social Services Team Manager, CXN a Connexions Worker and TC a Transitions Co-ordinator. Numbered suffixes identify specific individuals within these professional groups, *e.g.* TC-1).

**Extract 1:**


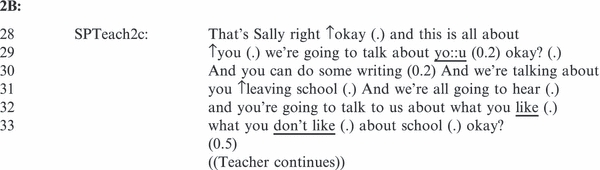


However, formally placing the young person at the centre of the process in this way is no guarantee of practical success. The extract below is an illustration of this, taken from a meeting in which initial introductions have just been carried out and a similar statement has been made to the one above:

**Extract 2:**


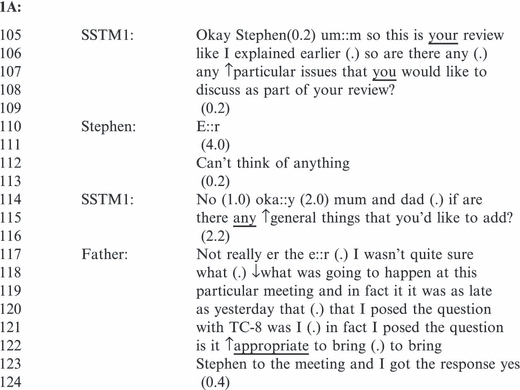


In this extract then, the young person has been placed at the centre of the review and is subsequently offered the chance (lines 106-08) to place items on the agenda. Following a pause and a filler (E::r) (lines 109-11), which suggest both that Stephen is having some difficulty formulating a reply and that his eventual answer is a dispreferred one (Pomerantz 1984a, Schegloff 2007) the opportunity is declined. The same opportunity (with a stress on any) is then offered to the young person's parents, who also decline, but with a reason for doing so. This extract makes explicit how difficult it can be to take control of an agenda ‘in the moment’, with only limited time in which to consider the issues. Both Stephen's pause-embedded ‘Can't think of anything’ and his father's report of being not ‘quite sure what (.) ↓what was going to happen’ (117-19) point to a situation in which they are considering this issue in the here and now. In one sense this may not be problematic, since issues can potentially be added to the discussion later on. However, by adding issues at a later point the speaker loses the interactional opportunity to shape the encounter from the outset. This scenario also runs the risk that the remit of the meeting is not clearly understood by all parties. In actual fact, in data not shown here, Stephen's father subsequently attempts to put an issue of transport on the agenda, only to be told that this is beyond the scope of the discussion. In other words, and as research in other institutional scenarios has suggested (*e.g*. Pilnick 2002), it may not be fruitful to offer interactional control *in* the meeting, without formulating a prior framework for that process.

## Questioning style

A related issue is that while a young person or a parent/carer may be free to put items on the agenda, there are a number of areas that *must* be covered, given that the endpoint of the process is to formulate a plan for transition. These items include what the young person is already doing and enjoying as part of their special education, as well as things they might like to do as part of a future plan. In this way the meeting has to cover past, present and future activities, activities at school home and college, as well as the aptitudes and abilities of the young person. As we shall see below, this wide-ranging remit can itself cause problems – given the wide range of referents that any question could relate to, it can sometimes be difficult to ascertain which is which.

### a) Past, present or future?

**Extract 3:**


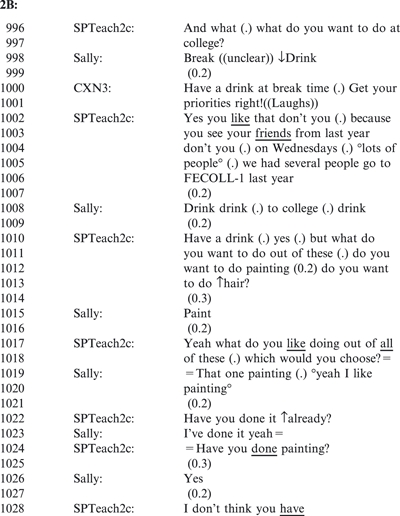


In this extract then, the teacher's initial question in line 996 is treated by Sally as a question about the present, and receives the response about having a drink at breaktime. Whilst the Connexions Worker treats this response in a jokey fashion (line 1000–01), the teacher at first takes it at face value, and subsequently provides an account for why Sally might particularly enjoy her breaks at college (1002–06). However, following Sally's restatement in line 1008, the teacher refines the question to ask ‘what do you want to do out of these’, indicating in lines 1011–12 a set of picture cards which represent various activities. Whilst ‘having a drink at breaktime’ may well be what Sally enjoys most about college, this clarification is presumably intended to receive an answer which is seen as adequate and appropriate for the purposes of the meeting rather than just interactionally adequate and appropriate. In a less formal context, the response might not need to be pursued further, but in a context geared towards further education, training or employment the appropriateness of an answer has also to be judged against its relevance to these topics.

The question ‘What do you want to do out of these’ (1010-11) is framed so that it might apply to the present or the future. ‘Painting’ is then offered as one of two specific possibilities and this is accepted by the young person in line 1015. The teacher marks receipt of this answer, but immediately asks an almost identical question, this time replacing ‘What do you want to do’ with ‘What do you like doing’ in line 1017. This latter question suggests an answer rooted in the present and an activity the student has some experience of, but the addition of ‘which would you choose’ in line 1018 serves to undermine any clarity or specificity as to whether this is intended to relate to activities that have already been done or that might be chosen in the future. In fact, the young person treats this question as a request for confirmation of her previous answer, and her response this second time (line 1019–20) is emphatic, using a pointing gesture as well as words. However, the teacher still does not treat this exchange as complete, and it is only the subsequent questions, ‘Have you done it ↑already’ (line 1022), and ‘Have you done painting’ (1024) that begin to make it clear that the previous question was intended to be heard as referring to past or present activities at *college* rather than possible future activities, or past activities elsewhere. As Pomerantz (1984b) notes, if a recipient fails to give what a questioner sees as a coherent response to a question, his or her behaviour is accountable, and the speaker makes sense of it in terms of the recipient having some problem in responding. Continuing questions, then, point to the perceived inadequacy of the previous answer. Through repair initiations, the teacher indicates that she has some trouble with Sally's answer, and twice provides her (lines 1023 and 1026) with an opportunity to put this right herself, before eventually providing an ‘other correction’ (Marlaire and Maynard 1990, Schegloff *et al.* 1977). In this case, then, repeated questioning causes confusion and leads to the ‘wrong’ answer from the teacher's point of view, since Sally has not yet studied painting at college, and only past college activities are deemed relevant to this section of the interaction. It is, however, a combination of lack of clarity from the questioner and misunderstanding from the recipient that leads to the production of an apparently inadequate answer. Put another way, the interactional troubles that arise here cannot be simplistically attributed to an inability to provide appropriate answers on Sally's part. Just as Sally's answer about having a drink at breaktime was interactionally appropriate but contextually problematic, so the issue over painting represents a confusion over context. While the young person may not have done painting at college, it is highly likely that she has ‘done painting’ of some kind at some point in her educational career, and is thus in a position to draw on this experience to appropriately state her enjoyment of it.

### b) Home, school or college?

A similar scenario of the young person providing an answer that is treated as ‘wrong’ or problematic for the purposes of the interaction at hand can be seen in the extract below, though this time the confusion is over location rather than time frame. Here, the teacher has immediately previously been asking Alec about activities he likes at school, though at the outset of the meeting she has said they will also talk about what he likes doing at home and would like to do at work or college.

**Extract 4:**


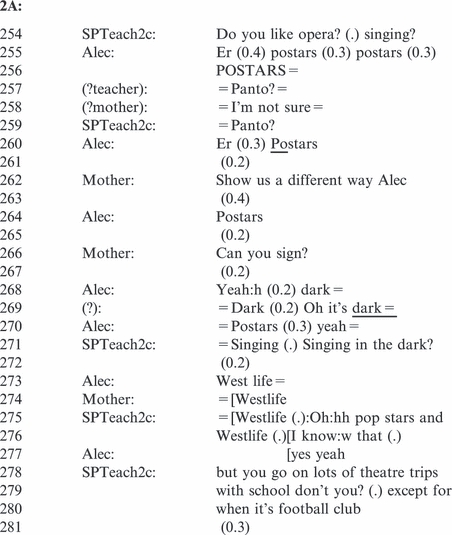


The first thing to note here is that the intonation used by the teacher in line 254 makes it potentially unclear whether this is to be heard as two questions (‘do you like opera’ and/or ‘do you like singing’) or one. The second is that, although this utterance ‘Do you like opera? (.) singing?’ is not framed specifically about whether the young person likes singing *at school*, it is clear from the teacher's ultimate response that this is how it is intended to be heard and responded to in this section of the discussion. Following the initial difficulty in interpreting Alec's response (lines 257–65), and his mother's suggestion (line 266) that he also sign his answer, which results in the signs for ‘darkness’ and ‘singing’, Alec eventually produces the utterance ‘Westlife’ in line 273 as a specific example of the general category he is trying to describe. Westlife are identifiable to the other people in the meeting as popstars and as a result Alec makes himself understood. Responding to the question ‘Do you like singing’ with ‘popstars’ and subsequently ‘Westlife’ is on the face of it an interactionally adequate and appropriate answer, since popstars and Westlife are indeed singers. The ‘Oh:hh’ that marks the start of the teacher's utterance here functions to display affiliation or alignment with Alec's answer, thereby confirming understanding (Heritage 1984b, 2002) but his subsequent talk in lines 276–79 both discounts it as newsworthy (‘I know that’) and treats it as inadequate, using ‘but’ to shift the focus to *school based* activities. Alec's participation in these school activities is formulated as potential evidence for a positive answer to the question about liking singing in this specific context. In other words, what Alec likes that is related to singing but *outside of school* may be an interactionally appropriate contribution but is not seen as contextually adequate at this time.

### c) Enjoyment or ability?

Obviously, anyone can enjoy an activity without having any particular aptitude for it (and indeed vice versa). This may be fine as it relates to activities enjoyed at home but is perhaps more problematic in terms of decisions as to appropriate college courses *etc*. Nevertheless, there appears to be some conflation between the two scenarios in some cases in this corpus, which serves as another instance of the complicated interactional contingenices that are at work here.

The example below is from Sally's review meeting, and illustrates a common occurrence in these data:

**Extract 5:**


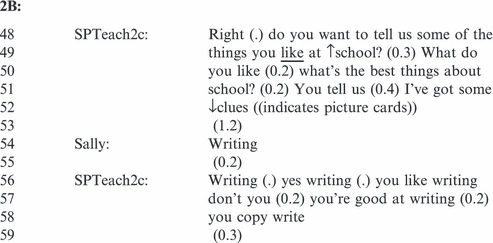


In this extract then, though the teacher's question at lines 48–52 is explicitly and repeatedly framed in terms of Sally's likes, the answer that she produces in line 54 – writing – is also evaluated in terms of her abilities. After acknowledging her enjoyment of the activity, the teacher produces the assessment ‘you're good at writing’ in line 57. The recurrence of this kind of formulation (‘Yes, you're good at that’) in the data when young people report their likes in a school or college context suggests that acceptance of an answer as appropriate may not depend solely on their preferences. This becomes apparent where a young person's enjoyment of and ability at an activity do not concur. The example below follows directly on from a lengthy series of questions about what Alec *likes* doing at school.

**Extract 6:**


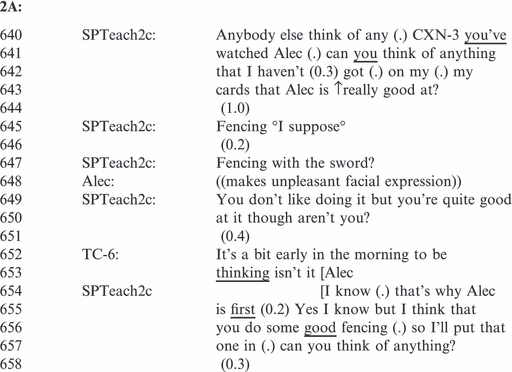


Where previous questions have been about liking activities, in the extract above, the participants are asked for the first time to think of something that the young person is good at. When the activity of fencing is raised by the teacher (lines 645–47), Alec's (non-verbal) response attends to the original question, indicating that he doesn't in fact like the activity. However, his response is noted by the teacher in line 649 but ultimately rejected, on the basis that he is ‘quite good at it’. Pomerantz (1984b) describes how speakers can move towards a pursuit of agreement by producing a series of evidential statements with which it is hard to disagree. Here, two types of evidence are presented – the first, repeated in lines 649 and 656 is that Alec is good at fencing. The second is that it is ‘a bit early in the morning to be thinking’ (lines 652-3) (this is the first meeting of the day), which seems to imply that Alec may not be able to think straight. The fact that Alec is good at fencing is ultimately used as a rationale for including fencing in the list of possibilities for future activities, despite his expressed resistance to this. In this case then, ‘is good at’ is allowed to stand in place of ‘likes’. This extract, then, highlights an extra level of complexity in these interactions. In Extract 4, Alec has been expected to frame his answer with reference to the previously invoked context of school, though the immediately prior question does not refer to this specifically. Here, his attempt to frame his answer with reference to the previously invoked context of enjoyment is disallowed.

## Questioning the answer: the consequences of ‘wrong’ answers

Clearly, staff in these review meetings face a very real difficulty in that they are attempting to discuss hypothetical future plans with young people whose communication skills are limited and whose responses may be at odds with the professionals’ experiences of working with them. This difficulty is alluded to in the extract below. Adam is a young person with extremely minimal verbal communication, some of which is seen as contradictory. The staff who are present are discussing (in his absence) how Adam's likes and dislikes can be warranted. They have been looking at photos of him engaged in various activities taken from his school record.

**Extract 7:**


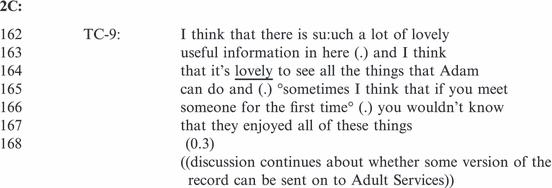


Here, the Transitions Co-ordinator makes explicit the dilemma for staff that arises from Adam's limited communication. She invokes the ‘evidence’ of the record to suggest ‘sometimes I think that if you meet someone for the first time you wouldn't know that they enjoyed all of these things’ (165-67), implying that the document provides a more adequate picture than could be gained from Adam himself, especially in the context of a one-off meeting with individuals who may be unfamiliar. As Finlay, Walton and Antaki (2008: 354) note in relation to their residential home data, ‘when understanding is uncertain and verbal communication limited staff have to decide whether a person is really exercising a choice, is simply choosing what they know or is responding to some feature of the options or content irrelevant to the choice being offered’. Evidently, in these scenarios, other factors may need to be brought to bear in determining a future course of action for the young person. However, the difficulty in this scenario is in judging the real ‘inappropriateness’ of a response. There are undoubtedly instances in these data where responses are unambiguously inappropriate – in an extract not shown here, for example, Louise cannot correctly answer where she regularly goes on a Monday. By contrast, Extracts 3, 4 and 6 above are instances where the response might come to be treated as inappropriate in this context, but as we have argued, they are interactionally appropriate answers which may arise as a result of ambiguity over the specifics of context. To cite Marlaire and Maynard (1990: 98), writing on test scenarios, ‘..interpretations of why a child is answering badly are embedded in the decision to accept some answers as ‘final’ and hence as correct or incorrect…’.

Returning to Rapley (2004) for a moment, he points out that one of the reported characteristics of acquiescence bias as a phenomenon is that people with ID respond ‘yes’ to contradictory questions (for example ‘Are you a man?’ and ‘Are you a woman?’). However, he highlights the difference between inconsistency and acquiescence, in the sense that in some cases it may not be the same question that is being asked, or at least that is understood to be asked, on different occasions. Extracts 3, 4 and 6 above, whilst largely using open questions rather than the closed questions Rapley focuses on in his analysis, illustrate such possible inconsistencies. What they also show is that, given the range of issues that need to be covered in these meetings, there is a very complex interactional process at work in the sense that each question has to be interpreted as referring to the intended overarching educational context, the intended time frame, the intended specific location, and that ability *or* enjoyment may be the key referent. Misinterpreting any of these may lead to inconsistent or apparently inappropriate answers. This has parallels with Maynard and Marlaire's (1992) observation in relation to testing for developmental disability, in which they draw on Lynch's (1984) work in neurobehavioural diagnosis, that failures during a collaborative process can be transformed into failures *of* an individual.

A crucial point to be made here, however, is that even outside a test scenario, these answers which are treated as ‘wrong’ by virtue of being seen as inappropriate in some way can have far reaching consequences. The extract below is taken from later in the meeting with Alec and begins immediately from the point at which he has left the room to return to his classroom.

**Extract 8:**


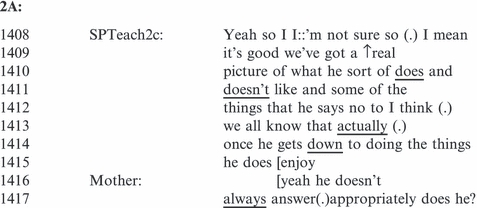


In this extract ‘so’ at the beginning of the teacher's utterance has the effect of connecting what follows to prior unfinished business, and marking it as a matter for ongoing concern (Bolden 2006). As it unfolds, the talk by SPTeach2c in lines 1408–1415 provides a rationale for continuing to focus on possibilities related to activities or future courses of action that the student has said he does not wish to pursue. This is achieved by appealing to collective knowledge (‘I think (.) we all know…’ in lines 1412–13), and the mother's overlapping utterance collaborates with this to discount some of her son's answers, though her stress on ‘always’ at the same time points up an underlying competency. In this example then, what has previously been seen as the young person's interactionally inappropriate answering (for example Extract 6 above) is used as a rationale for keeping open options he has expressed resistance to. However, these ‘inappropriate’ responses have to be seen in the light of the ambiguous frame for interpreting questions we have highlighted in previous examples.

A similar kind of rationale is used towards the end of Sally's review, after she has left the room, when a further meeting is proposed to consider some options Sally has previously verbally expressed resistance to:

**Extract 9 :**


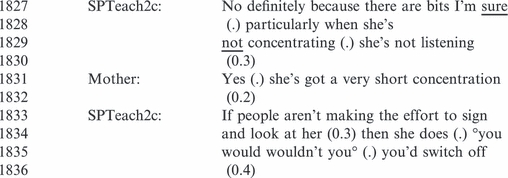


Again in this extract the teacher presents a view of the young person as an imperfect participant (lines 1827–29) which is then reinforced by her mother and which is ultimately used as a rationale for pursuing the further meeting. Interestingly, in this case the teacher presents Sally's failure to concentrate as understandable in the circumstances, given her limited communication skills (lines 1833–35) – there is no blame on Sally's part since this is what anyone would do. Following Finlay, Antaki and Walton (2008), we note that the teacher's utterance puts a positive gloss on Sally's behaviour, and suggests that this is a known part of her disposition. Nevertheless, Sally's communication failures are still used as the justification for overriding her views, despite the fact that we have seen in Extract 3 that some of these ‘failures’ may merit closer examination.

## Rejecting the young person's wishes – the limits of self-determination

The final extracts to be examined here also relate to instances where the young person's wishes or desires are rejected. We suggest, however, that these form a different analytic category. Whereas in Extracts 3, 4 and 6 above the young person's expressed views are overridden on the basis of being interactionally inappropriate, because they attend to referents other than the intended ones, in the examples below they are overriden because they are inappropriate in a wider sense. Extracts 10 and 11 are taken from Alec's Transition Review Meeting, where he has stated at the very outset that he would like to join the police. As we shall see, the participants avoid directly suggesting that this may be beyond his competency.

**Extract 10:**


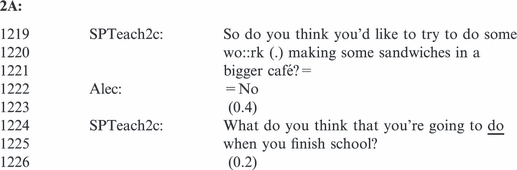



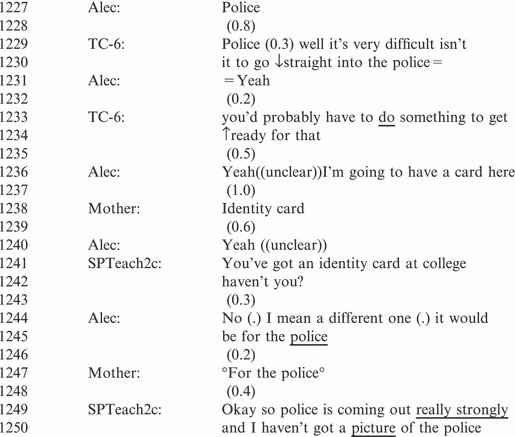


Once again in this extract, the teacher's use of ‘so’ connects the proposal that follows to previous unfinished business which remains a concern (Bolden 2006). The teacher's proposal concerns making sandwiches (which Alec has some experience of, and which could form the basis of paid work) and is explicitly rejected by Alec in line 1222. The teacher's subsequent question, with its emphasis on ‘do’, attends to one function of the meeting being to settle on a purposeful and focused plan for Alec which incorporates further education, training, work or placement with a day service, but Alec responds to this question by clearly stating again his desire to work for the police in line 1227. TC-6’s response, beginning at line 1229, refers obliquely to the fact that this is unlikely, but doesn't reject the young person's expressed desire in any definitive way, saying that it will be ‘very difficult’ and that the young person will ‘probably’ have to do something else first. The indirectness of the utterance hints at its dispreferred nature (Pomerantz 1984a), and it shares some characteristics with features of bad news delivery (Maynard 2003) in that it is delayed by opinion markers and then hinted at rather than produced explicitly. It seems likely that the very delicate and indirect rejection of this desire is related to the wider delicacy over young people's competencies that pervade these encounters. However, the end result is that, in this section of the discussion, joining the police as a possible future course of action is not overtly rejected. Some more discussion follows (about the young person's competency with keys, and the need to consider possible college placements) and then the conversation continues as follows as the teacher moves to bring the section of the meeting with Alec in attendance to a close.

**Extract 11:**


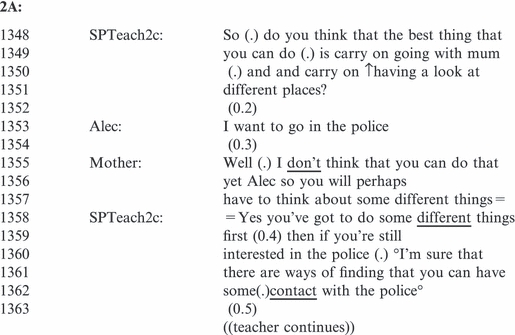


The teacher's suggestion at this point is that Alec should continue to look at possible colleges, sites of employment etc with his parents, before making a final choice. In response to this, Alec once again clearly states his desire to join the police (line 1353). The response to this, this time from his mother in line 1355–57, is once again tentative, hedged with ‘I don't think’ and ‘you will perhaps have to think’. Her use of ‘yet’ has the effect of rejecting this proposal only for the present time, rather than for ever. This temporal aspect of the rejection is echoed in the teacher's last utterance, so although it provides for a more definitive rejection –‘you've *got to* do some different things first’ (our emphasis) (1358–59), it is still not rejected out of hand.

This extract is interesting because, where it might be argued that previous extracts have shown a lack of sensitivity to students’ expressed wishes or choices, this shows the opposite. The young person's choice, though outside his current competency, is treated with the utmost delicacy. On a number of occasions, its rejection is so delicate that it may be difficult to understand these as rejections at all. MacIntyre (1999) argues that two of the three elements of the capacity to be an ‘independent practical reasoner’ are the ability to distance oneself from immediate desires and recognise that these may not coincide with one's best interests; and the recognition of the possibility of alternative realistic futures. We would suggest that it is precisely these abilities, or the lack of them, that are at stake here. Hence the delicacy of the rejection is unsurprising, given that its wider implication is that Alec is unable to discern his own best interests, which is difficult territory for ID professionals in the context of the dominant discourse of self-determination and choice. However, the practical interactional consequences of this delicacy are that Alec continues to pursue the proposal until a considerable amount of interactional work is required to close it down.

## Conclusions

The current trend in ID-specific policy is towards independence and choice, as part of a wider policy trend towards empowerment through self-sufficiency. However, it is not unproblematic to offer these in an ID context, particularly one which is as interactionally complex as the Transition or Leaver's Review Meetings reported here. Redley and Weinberg's (2007) study of interaction in the Parliament for People with Learning Disabilities offers a critique of much previous interactional work on ID, saying that it implicitly accepts a liberal model of citizenship that requires intellectual ability and independence in order to make choices. They point to the paradox that the entitlement of people with intellectual disabilities to assistance from the state depends on their ID, and yet they are assumed to be empowered through liberal citizenship. In their data, they point to examples of where ‘giving the floor’ to persons with ID fails to work, and what follows is a kind of classroom exchange in which answers have to be dragged out by use of leading questions. As a result, they argue that in attempting to allow autonomy, we risk falling back on old practices of ‘educating’ ID people. Similarly, Finlay, Walton and Antaki (2008) note that people with ID are in particular danger of being viewed indefinitely as people who lack ‘capacity’ in some sense, and as a result will always be cast as needing further training or teaching. We have seen some examples of a similar sort of process in these data, for example in the opening extracts where the floor, though offered, is not taken, and in Extracts 3 and 4 where the ‘right’ answer is eventually achieved through continued questioning. However, what we are arguing here goes beyond a critique of interactional practices of ‘educating’. We suggest that an attempt to be empowering can actually and paradoxically end in an undermining of choice and control. When, in the complex interactional circumstances of Transitions, young people are seen to fail to make appropriate decisions themselves, the decisions are then made for them by others, sometimes once the young people have left the room. The problem is that, in line with policy goals, young people in this sample begin by being treated as unproblematically competent to manage their contribution to these meetings, both in interactional terms and in terms of their capacity as ‘independent practical reasoners’ (MacIntyre 1999). In practical terms, an interactional construction of competence at the outset is necessary for the meeting to take place at all, as without this it would have no legitimacy. However, if the young people ‘fail’ in some aspect of interactional practice (*e.g*. failure to understand that a question specifically relates to the future rather than the past or present), this failure can be taken to cast doubts on whatever they have said, and subsequently used to override wishes or feelings that may have been quite clearly expressed. In other words, answering a question in a way that is perceived as inadequate can lead to questioning of other answers. This leads to specifically interactional impediments to autonomy. At the same time, an attempt to preserve autonomy can create interactional difficulties of its own, since rejection of the young person's stated wishes must be so delicately managed. This latter scenario provides practical demonstration of Fisher's (2008) assertion that a focus on empowerment tends to underplay constraints.

An obvious consequence of these findings is that staff are put in an impossible situation. On the one hand, they are trying to offer client-centred interactions that place the young person's competency and their right to express their desires at the heart of the transitions process. They carry responsibility for enabling the young person to take part and express choices. On the other, they are tasked with managing the fact that a lack of competency on the part of the young people in question means that not all of these desires, however clearly expressed, can be translated into reality. What is being asked of staff is undeliverable; policy leads them to a position that they cannot maintain. In addition, they must manage these tensions within a wider welfare policy context which promotes guiding the young people towards realistic types of employment or training, and which places an emphasis on finding work that an individual is capable of doing rather than work they might aspire to or imagine they would enjoy. Finlay, Walton and Antaki (2008) give a compelling account of how staff in residential settings are torn between being a good keyworker (for example, providing choice) and a good employee (for example, getting residents fed), as well as being representatives of their profession. This latter point is of particular salience to the data presented here, since all the professionals involved have an immediate audience of other professionals to their conduct. As a result, failure to manage conflicting demands risks staff's *own* competency being brought into question.

In terms of the wider implications of this work, we have illustrated some of the ways in which policy directions can impact on day-to-day interactional practice between young adults with intellectual disabilities and those professionals involved in their care. More specifically, *Valuing People* suggests that the principle of self-determination can be universally applied to achieve empowerment. Here we have seen the interactional and practical difficulties that result from an attempt to carry this through.We recognise, however, that any step away from this would deny professionals both the shelter of a rule-based approach to practice and the moral clarity associated with asserting client empowerment as a service goal, despite the fact that the end result does not necessarily achieve these aims. A more discretionary approach might be more practically useful, and interactionally easier to manage, but it would also risk exposing professionals’ failure to reconcile potentially contradictory imperatives to the gaze of their peers.
